# Glutamine and glutaminolysis are required for efficient replication of infectious spleen and kidney necrosis virus in Chinese perch brain cells

**DOI:** 10.18632/oncotarget.13681

**Published:** 2016-11-29

**Authors:** Xiaozhe Fu, Xianqin Hu, Ningqiu Li, Feifei Zheng, Xingxing Dong, Jing Duan, Qiang Lin, Jiagang Tu, Lijuan Zhao, Zhibin Huang, Jianguo Su, Li Lin

**Affiliations:** ^1^ Pearl River Fisheries Research Institute, Chinese Academy of Fishery Sciences, Key Laboratory of Fishery Drug Development, Ministry of Agriculture, Key Laboratory of Aquatic Animal Immune Technology, Guangdong Provinces, Guangzhou, Guangdong, 510380, China; ^2^ Department of Aquatic Animal Medicine, Research Center of Marine Biology, College of Fisheries, Freshwater Aquaculture Collaborative Innovation Center of Hubei Province Huazhong Agricultural University, Wuhan, Hubei, 430070, China; ^3^ School of Animal Sciences and Nutritional Engineering, Wuhan Polytechnic University, Wuhan, Hubei, 430023, China; ^4^ College of Animal Science and Technology, Northwest A and F University, Shanxi Key Laboratory of Molecular Biology for Aquaculture, Yangling, 712100, China; ^5^ State Key Laboratory of Marine Resource Utilization in South China Sea, Hainan Provincial Key Laboratory for Tropical Hydrobiology and Biotechnology, College of Marine Science, Hainan University, Haikou 570228, China

**Keywords:** Siniperca chuatsi, ISKNV, glutamine, glutaminolysis, TCA cycle

## Abstract

Viruses rely on host cellular metabolism for energy and macromolecule synthesis during their replication. Infectious spleen and kidney necrosis virus (ISKNV) causes significant economic losses in the Chinese perch (*Siniperca chuatsi*) industry worldwide. However, little is known about the relationship between ISKNV replication and cellular metabolism. Using transcriptomic analysis, we observed that glutamine metabolism in Chinese perch brain (CPB) cells is altered during ISKNV infection. Moreover, ISKNV replication was decreased in CPB cells cultured in the glutamine-depleted medium. ISKNV replication was also inhibited in CPB cells cultured in the presence of bis-2-(5-phenylacetamido-1,3,4-thiadiazol-2-yl) ethyl sulfide (an inhibitor of glutaminase), (–)-epigallocatechinmo nogallate (an inhibitor of glutamate dehydrogenase) or L-buthionine sulfoximine (an inhibitor of glutathione synthesis). However, virus replication was rescued by the addition of multiple tricarboxylic acid cycle intermediates, ATP, or glutathione reduced ethyl ester. ATP and reduced glutathione/oxidized glutathione levels were increased in CPB cells infected with ISKNV, but were decreased in CPB cells cultured in glutamine-depleted medium. These results indicate ISKNV infection induces glutaminolysis to accommodate the biosynthetic and energy needs for its efficient virus replication.

## INTRODUCTION

Viruses hijack host cellular machinery to facilitate its own replication. Many viruses, including human cytomegalovirus (HCMV) [1, 2] Kaposi's sarcoma-associated herpesvirus (KSHV) [3], herpes simplex virus 1 (HSV-1) [4], hepatitis C virus (HCV) [5], human immunodeficiency virus (HIV) [6], dengue virus [7], and white spot syndrome virus (WSSV) [8, 9], induce multiple cellular metabolic alterations. For example, HCMV up-regulates fatty acid synthesis, pyrimidine nucleotide biosynthesis, and glycolysis [1, 2].

Glutamine is an abundant amino necessary generation of energy and synthesis of macromolecules in cells [10]. For energy production, glutamine is initially oxidized to glutamate in a reaction catalyzed by glutaminase. Thereafter, glutamate dehydrogenase (GDH) catalyzes the conversion of glutamate to α-ketoglutarate (α-KG), which enters the tricarboxylic acid (TCA) cycle. During glutaminolysis, glutamine provides a variety of important TCA intermediates, including the reduced form of nicotinamide adenine dinucleotide (NADH) for oxidative phosphorylation, succinyl coenzyme A (CoA) for adenosine 5ʹ-triphosphate (ATP) generation, and glutathione precursor (glutamate). Glutamine is also essential for the infection and replication of HIV [11], HCMV [10], and vaccinia virus (VACV) [12]. However, the underlying mechanisms remain unclear.

Infectious spleen and kidney necrosis virus (ISKNV) is the type species of the genus *Megalocytivirus* in the Iridoviridae family [13], which is highly lethal in Chinese perch (*Siniperca chuatsi*) and can cause great economic losses [14, 15]. A survey on the host ranges of ISKNV showed that the virus can infect more than 50 marine and freshwater fish, including the species in the *Perciformes*, *Pleuronectiformes*, *Clupeiformes*, *Tetraodontiformes*, *Myctophiformes*, and *Mugiliformes* orders [16]. At present, ISKNV is one of the most important causative agents of fish disease and is listed by the International Epizootic Office (OIE). However, there is currently no vaccine or no other strategies to effectively prevent ISKNV infection. Therefore understanding the relationship between ISKNV replication and cellular metabolic pathways could pave a new way for effective new prevention strategies against the ISKNV infection. Unfortunately, a global metabolic analysis of cells infected with ISKNV is not available.

To identify alterations of host cellular metabolic pathways during ISKNV infection, we analyzed the transcriptomic profile of healthy and ISKNV-infected CPB cells. Our results show that for efficient ISKNV replication, exogenous glutamine is required to replenish TCA cycle intermediates and the ATP supply and for glutathione synthesis.

## RESULTS

### ISKNV infection altered glutamine metabolism

**Figure 1:**
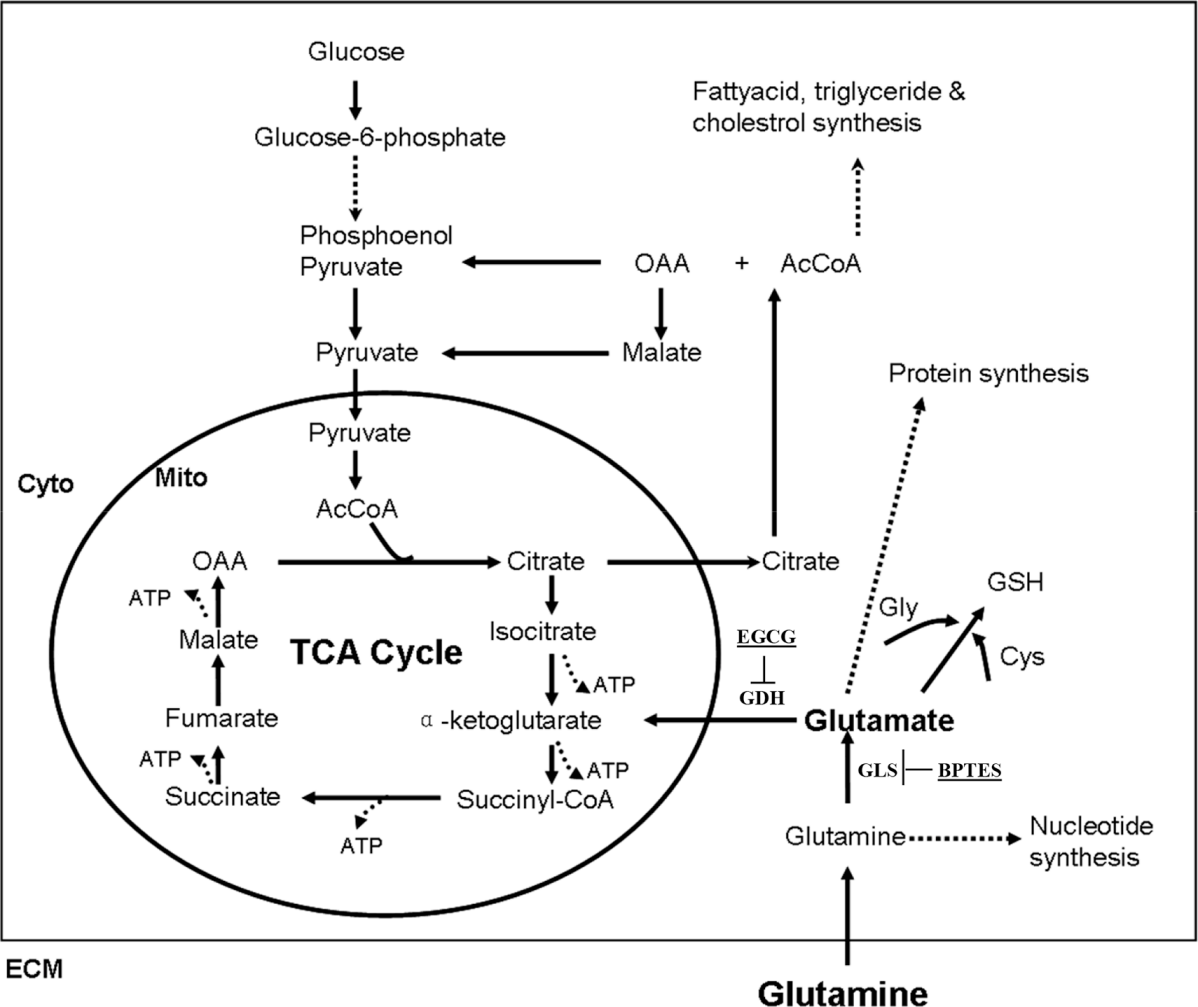
Simplified schematic of glutamine metabolism Reactions within the box are intracellular, and circle occurs in the mitochondrion (Mito), whereas those outside in the circle occur in the cytoplasm (Cyto). Dashed lines indicate that there are several intermediates formed (several reactions) between the ones shown. Abbreviations: TCA cycle (tricarboxylic acid cycle); OAA (oxaloacetic acid); AcCoA (acetyl coenzyme A); GSH (glutathione); Gly (glycine); Cys (cysteine); ECM (extracellular matrix).

Glutamine is metabolized to replenish the TCA cycle intermediates, meanwhile produces ATP via glutaminolysis (Figure 1). To investigate the effects of ISKNV infection on glutamine metabolism, we have analyzed the transcriptomic profiling of CPB cells infected by ISKNV [17]. Interestingly, ISKNV could induce the up-regulation of the mRNA expressions of some enzymes involved in glutamate metabolism, TCA cycle, and glutathione metabolism (Table 1), indicating that glutamine metabolic pathways might be altered in CPB cells infected with ISKNV.

**Table 1:** List of major enzymes involved in glutamine metabolism by transcriptomic analysis of chinese perch brain cells infected with infectious spleen and kidney necrosis virus

Pathways	Gene names	log2 Ratio (24hV/24hC)	log2 Ratio (72hV/72hC)
glutamate metabolism	Glutaminase (GLS)	2.79	1.44
	Glutamate dehydrogenase mitochondrial-like (GDH)	1.04	0.82
	Glutaminase kidney isoform	11.9	1.44
TCA cycle	Phosphoenolpyruvate carboxykinase (PECK)	1.20	0.73
	Glucose-6-phosphate 1-dehydrogenase-like (G6PD)	11.14	9.17
	Isocitrate dehydrogenase [NAD] subunit alpha (IDH)	11.21	7.64
	Succinate dehydrogenase [ubiquinone] iron-sulfur subunit (SDH)	1.32	0.72
Glutathione metabolism	3-hydroxyisobutyrate dehydrogenase (HIBADH)	1.77	–0.78
	Glutamate—cysteine ligase catalytic subunit (GCLC)	1.34	0.28
	Glutathione synthetase-like isoform 1 (GSHB)	4.05	–1.14

Note: CPB cells were infected with ISKNV at a MOI of 1. Cells infected with or without ISKNV were harvested at 24 and 72 hpi for transcriptomic analysis. V ISKNV; C, control.

### Glutamine was not essential for the viability of CPB cells, but it was required for efficient ISKNV multiplication

**Figure 2:**
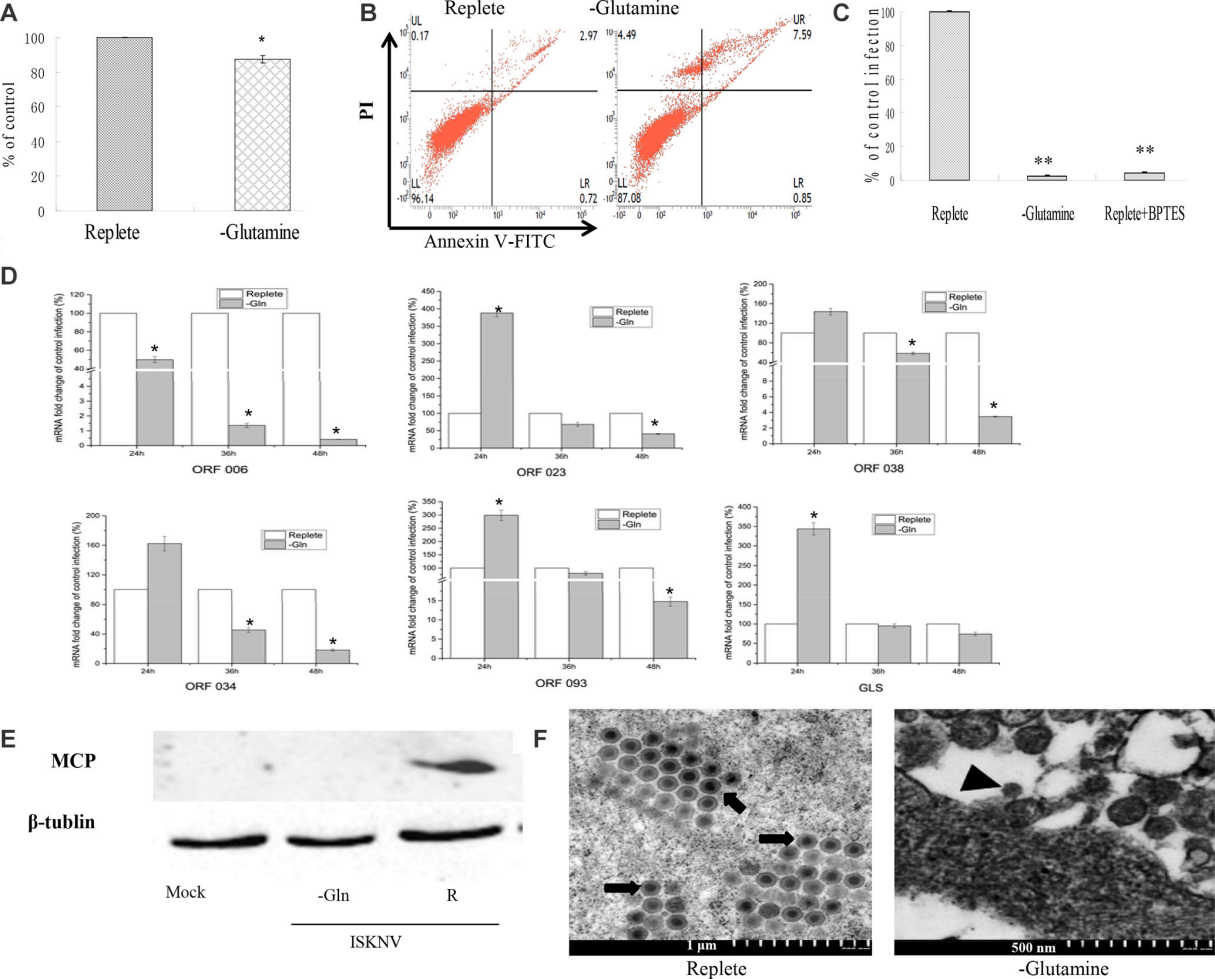
Glutamine is necessary for optimal infectious ISKNV production (**A**) Cell viability of CPB. Cells were fed with repleted medium or glutamine-depleted medium, respectively. At 72 h post treatment, cell viability was measured by MTS assay. (**B**) Apoptosis of CPB cells. The apoptosis of CPB cells cultured with glutamine or without glutamine for 72 hours was determined by AnnexinV-FITC/PI staining combined with flow cytometry assay. (**C**) Comparison of viral production. CPB cells were infected with ISKNV at an MOI of 1 and fed replete medium, glutamine-depleted medium, or fed replete medium with 10 μM BPTES at 2 hpi. The supernatant was harvested at 72 hpi, and viral yields were determined by TaqMan q-PCR. (**D**) Quantitative real-time RT-PCR analysis of ORF023, ORF038, ORF034, ORF093, ORF006 and GLS transcript levels in ISKNV-infected cells supplemented with or without glutamine. (**E**) Immunoblot analysis of MCP levels in ISKNV-infected cells fed replete medium (R), or glutamine-depleted medium (-Glu). Lysates from cells harvested at 72 hpi were subjected to western blot analysis using antibodies against MCP and loading control β-tublin. (**F**) Transmission electron micrograph analysis of virion formation in ISKNV-infected cells cultured with or without glutamine medium. The arrows show mature virion; the arrow head shows immature virion particles. *p < 0.05; **p < 0.01.

The CPB cells were cultured in the DMEM medium without glutamine for 72 hours, and the viability of CPB cells was about 92% compared with that cultured in glutamine repleted-medium (Figure 2A), indicating that glutamine was not essential for the viability of CPB within 72 h. To further determine whether the absence of glutamine affected physiological function and resulted in the apoptosis, AnnexinV-FITC/PI staining combined with flow cytometric analysis was used to detect the apoptosis. Compared with control cells cultured with glutamine, lack of glutamine resulted in a slight increase of apoptosis cell percentage from 2.97% to 7.95%, and a slight decrease of the live cell percentage from 96.14% to 87.08% (Figure 2B), indicating that lack of glutamine had no significant effect on the apoptosis and survival of CPB within 72 h.

Subsequently, we investigated the necessity of glutamine for ISKNV multiplication. As shown in Figure 2C, lack of glutamine reduced the yield of ISKNV around 97.85%. Furthermore, addition of BPTES (an inhibitor of glutaminase) into the glutamine-replete medium reduced ISKNV yield around 96.67%, indicating that glutaminolysis was required for the efficient ISKNV multiplication.

To identify the stage-wise requirement of glutamine in the ISKNV life cycle, we examined the viral mRNA transcription, viral protein synthesis, and virion formation in the CPB cells cultured in the medium supplemented with or without glutamine. It has been shown that ISKNV ORF023 and ORF038 were the immediate early genes, ORF034 and ORF093 were early genes, and ORF006 (major capsid protein gene, MCP) was the late gene of ISKNV [17]. Figure 2D showed that in the CPB cells cultured in glutamine-depleted medium, ORF006 mRNA synthesis was inhibited at either time point post-infection. However, the mRNAs of ORF023, ORF038, ORF034 and ORF093 were increased at 24 hours post-infection (hpi). Subsequently, they were decreased at 36 and 48 hpi, indicating that glutamine deprivation altered the viral transcription. Glutaminase is the enzyme which catalyzes the conversion of glutamine to glutamate by glutaminolysis. Therefore, the mRNA of glutaminase was monitored and results showed that it was increased at 24 hpi, subsequently was decreased at 36 hpi and 48 hpi. The expression pattern of glutaminase was similar to the expressions of immediate early gene and early gene of ISKNV. Next, we examined the ISKNV MCP protein expression. As shown in Figure 2E, MCP synthesis was blocked when ISKNV-infected cells were cultured in glutamine-depleted medium, suggesting that exogenous glutamine was necessary for ISKNV protein synthesis. To further investigate whether glutamine deprivation had an impact on ISKNV maturation, the virion formation was observed by transmission electron microscopy (TEM). Figure 2F showed that a large number of ISKNV particles were observed in glutamine-replete infected cells at different stages, but only little immature ISKNV particles were observed in glutamine-depleted infected cells. This result clearly indicated that glutamine was required for ISKNV virion maturation in CPB cells. Taken together, these experiments showed that glutamine was required for ISKNV multiplication.

### Glutamine was necessary for ISKNV multiplication via maintaining TCA cycle

**Figure 3:**
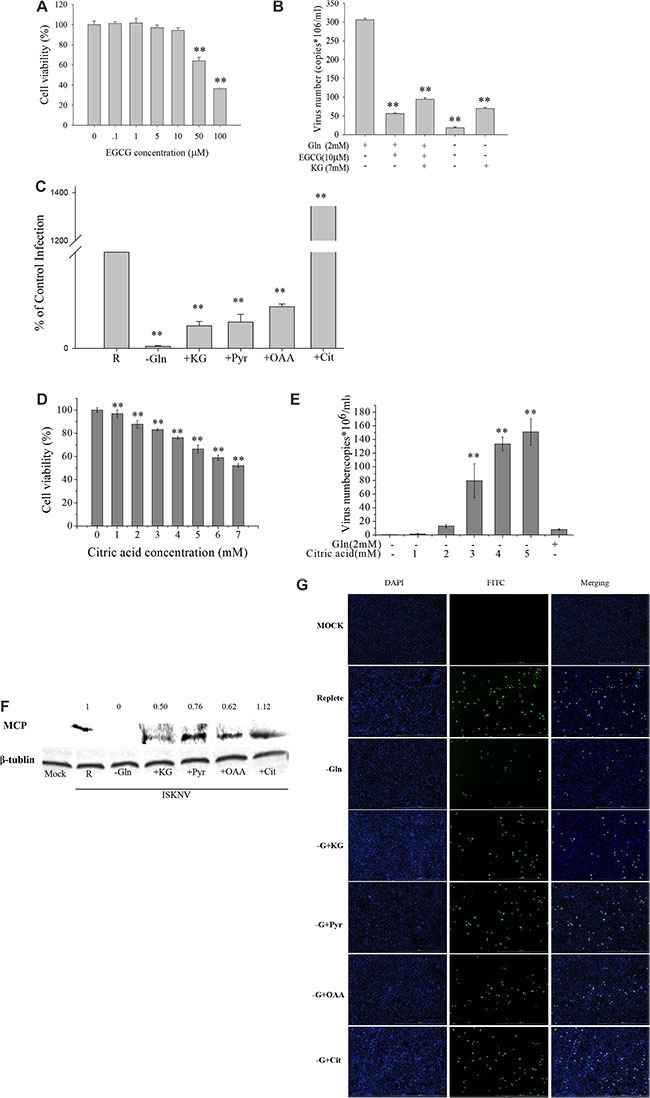
Glutamine is an essential anaplerotic substrate for the TCA cycle during ISKNV infection (**A**) CPB cells fed with repleted medium were treated with different concentrations of EGCG for 72 h, and the cell viability was measured by MTS assay. The activity of mock-treated samples was assigned as 100%. (**B**) CPB cells were infected with ISKNV at an MOI of 1 and were fed replete medium, or replete medium supplemented with EGCG, or replete medium supplemented with EGCG and 7 mM dimethyl-α-ketoglutarate (α-KG), or glutamine-depleted medium (-Glu), or glutamine-depleted medium supplemented with α-KG (7 mM). ISKNV from the cell supernatant was harvested at 72 hpi, and viral yield was determined by TaqMan q-PCR. (**C**) CPB cells were infected with ISKNV at an MOI of 1 and were fed replete medium (R), glutamine-depleted medium (-Glu), or glutamine-depleted medium supplemented with 7 mM dimethyl-α-ketoglutarate (α-KG), 4 mM pyruvate (Pyr), 4 mM oxaloacetic acid (OAA) or 4 mM citric acid-anhydrous (Cit) at 2 hpi. Cell-associated virus was harvested at 72 hpi, and viral yields were determined by TaqMan q-PCR. (**D**) CPB cells fed with glutamine-depleted medium were treated with different concentrations of citric acid for 72 h, and the cell viability was measured by MTS assay. The activity of mock-treated samples was assigned as 100%. (**E**) CPB cells in the presence or absence of glutamine or citric acid were infected with ISKNV at an MOI of 1 at 2 hpi. Cell supernant was harvested at 48 hpi, and viral yield was determined by TaqMan q-PCR. (**F**) Immunoblot analysis of ISKNV MCP levels in ISKNV-infected cells fed the different medium same as B. Lysates from cells harvested at 72 hpi were subjected to western blot analysis using antibodies against MCP and loading control β-tublin. The values up the lanes indicated the relative intensity of each major band. (**G**) Immunofluorecent analysis of MCP levels in ISKNV-infected cells fed the different medium same as C. **p < 0.01.

Glutamine is the primary source of carbon for energy homeostasis and biosynthesis in cells. Our transcriptomic results showed that ISKNV could induce multiple metabolic alterations, including up-regulation of the mRNA expressions of some enzymes involved in TCA cycle (Table 1). This prompted us to investigate whether ISKNV-infected cells required glutamine to maintain TCA cycle. Glutamate dehydrogenase (GDH) catalyzes glutamate to α-ketoglutarate which enters to TCA cycle [18], and EGCG is a specific inhibitor of GDH [19]. Figure 3A showed that the viability of CPB cells was 94.26% in the presence of 10 μM EGCG, but viral production was significantly reduced by 81.71% when cells cultured with glutamine-repleted medium were treated with 10 μM EGCG (Figure 3B). Supplement with α-KG (7 mM) in ISKNV-infected cells cultured with glutamine-repleted medium supplemented with EGCG could increase the ISKNV yield by approximately 68.5% (Figure 3B), indicating that a part of glutamine was catalyzed to α-ketoglutarate which entered to TCA cycle to rescue ISKNV production. When TCA cycle intermediate (including α-KG, oxaloacetic acid (OAA), pyruvate, and citric acid) was added to the medium of glutamine-depleted ISKNV-infected cells, respectively, Figure 3C showed that virus productions in cells cultured in glutamine-depleted medium supplemented with TCA cycle intermediates were significantly higher than that in glutamine-depleted cells. Interestingly, the yield of the virus was increased in the cells cultured in the medium supplemented with citric acid in a dose dependent manner (Figure 3D and 3E). As shown in Figure 3F and 3G, viral MCP expression was suppressed in CPB cells cultured in glutamine-depleted medium, but it was substantially recovered by the additional supplementation of TCA cycle intermediates (α-KG, pyruvate, OAA or citric acid). Taken together, these results showed that exogenous glutamine served as an anaplerotic substrate replenishing the TCA cycle to partly rescue ISKNV production.

### Glutamine supported efficient ISKNV multiplication also by supplying ATP

**Figure 4:**
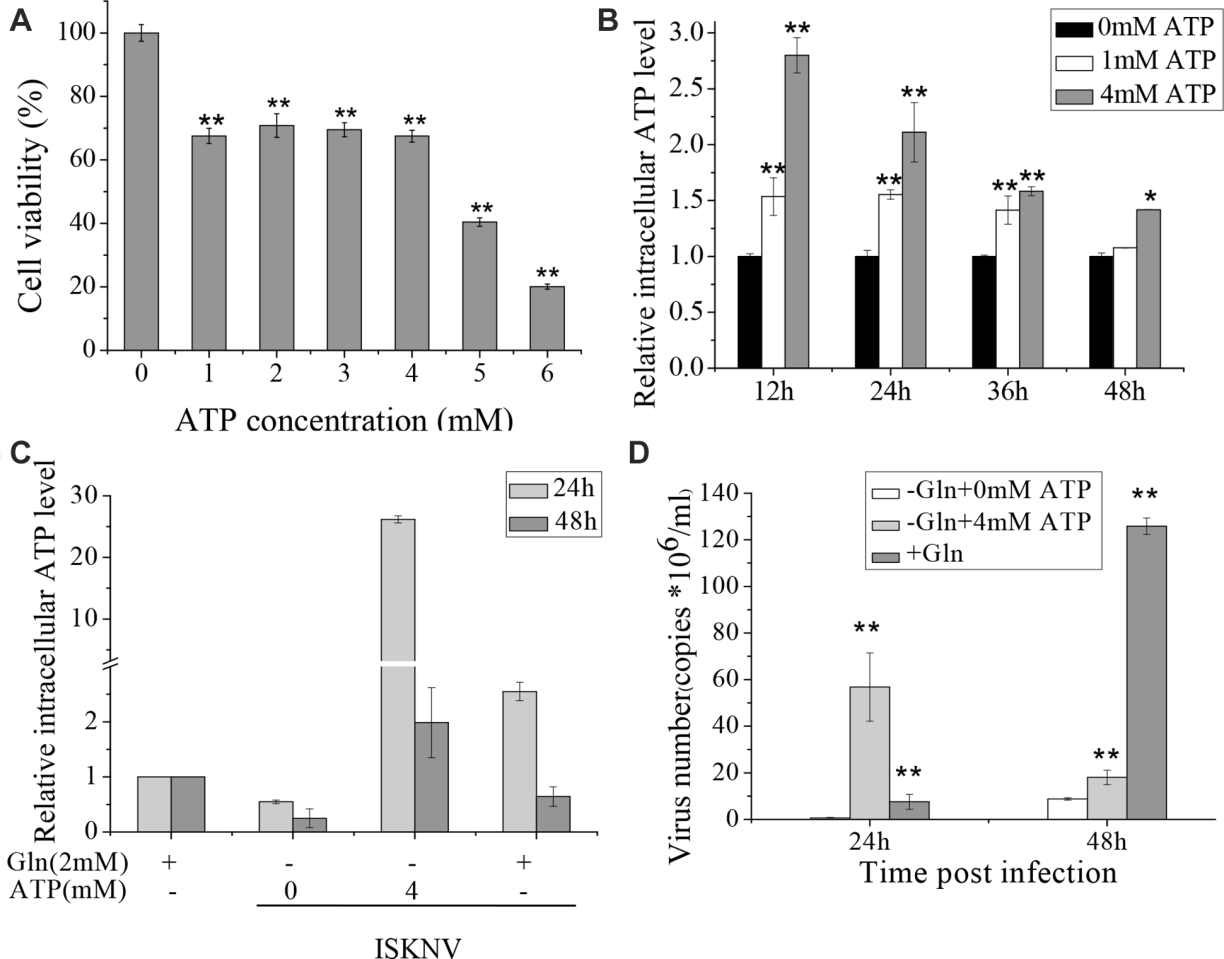
Glutamine provides ATP to induce the rapid ISKNV release (**A**) CPB cells fed with glutamine-depleted medium were treated with different concentrations of ATP for 72 h, and the cell viability was measured by MTS assay. The activity of mock-treated samples was assigned as 100%. (**B**) Intracellular ATP levels in CPB cells treated with ATP of 0, 1, 4 mM were determined by ATP Colorimetric/Fluorometric Assay Kit. The ATP concentration of mock-treated samples was assigned as 1. (**C**) Intracellular ATP levels of CPB cells infected with ISKNV at an MOI of 1 at 2 hpi in the presence or absence of glutamine or ATP. Intracellular ATP levels were examined at 24 and 48 hpi, and the level of cells without ISKNV infection was set as 1. (**D**) CPB cells in the presence or absence of glutamine or ATP were infected with ISKNV at an MOI of 1 at 2 hpi. ISKNV of cell supernatant was harvested at 24 and 48 hpi, and viral yield was determined by TaqMan q-PCR. *p < 0.05; **p < 0.01.

Besides replenishing the TCA cycle intermediates, glutamine also produces ATP via glutaminolysis. There is growing evidence that ATP is an energy source for maturation and assembly of many enveloped viruses [20, 21]. To investigate whether ISKNV multiplication in CPB cells required glutamine to produce ATP, glutamine or ATP was added to the medium of glutamine-depleted infected cells. CPB cell viability was measured when the cells were cultured in glutamine-depleted medium supplemented with ATP. The result showed that cell viability was not significantly decreased when CPB cells were cultured in glutamine-depleted medium supplemented with additional ATP at 1, 2, 3, 4 mM (about 75% of that in the glutamine repleted medium). However, it was reduced to about 40% in glutamine-depleted medium supplemented with additional 5 or 6 mM ATP (Figure 4A). When additional 1 or 4 mM ATP was added to CPB cells cultured in glutamine-depleted medium, the intracellular ATP levels were significantly increased until 48 hours (Figure 4B), indicating that addition of ATP in the medium could enhance the intracellular ATP concentration. Moreover, when CPB cells were cultured in medium supplemented with glutamine, the intracellular ATP was enhanced (Figure 4C), indicating that addition of glutamine was directly related with the intracellular ATP production. Thus, we further investigated the relationship between the multiplication of ISKNV and intracellular ATP production. The result showed that the addition of ATP was able to increase the yield of ISKNV in the supernatant of CPB cells cultured in glutamine-depleted medium (Figure 4D), indicating that supplying ATP for efficient ISKNV replication was also a role of glutamine.

### Glutamine provided substrates for glutathione synthesis to promote ISKNV multiplication

**Figure 5:**
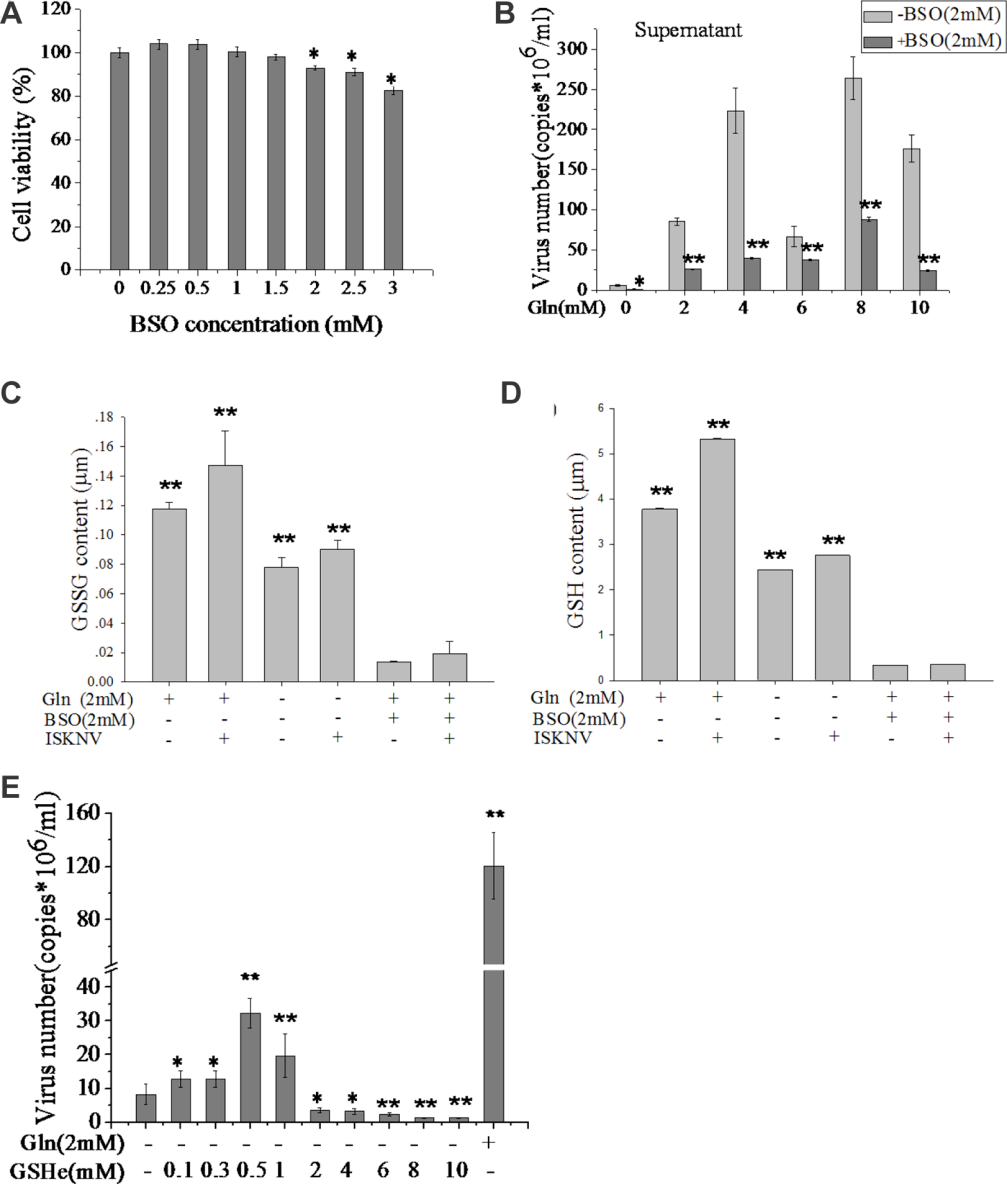
Glutamine provides substrates for GSH synthesis to promote ISKNV replication (**A**) CPB cells fed with glutamine medium were treated with different concentrations of BSO for 72 h, and the cell viability was measured for MTS assay. The activity of mock-treated samples was assigned as 100%. (**B**) CPB cells in the presence or absence of BSO (2 mM) and various concentrations of glutamine were infected with ISKNV at an MOI of 1 at 2 hpi. ISKNV of cell supernatant was harvested at 48 hpi, and viral yield was determined by TaqMan q-PCR. (**C**) Comparison of GSSG and (**D**) GSH content of CPB cells in the presence or absence of glutamine, BSO, or ISKNV infection. (**E**) CPB cells in the presence or absence of glutamine or GSHe were infected with ISKNV at an MOI of 1 at 2 hpi. Cell supernatant was harvested at 48 hpi, and viral yield was determined by TaqMan q-PCR. *p < 0.05; **p < 0.01.

Glutathione consists of glutamate, cysteine, and glycine. It has been shown that glutathione storage correlated with the loss of glutamine and glutamate, but not with cysteine or glycine [22, 23]. Thus we examined the influence of glutathione on ISKNV replication. L-Buthionine sulfoximine (BSO) is a specific inhibitor for inhibiting the synthesis of glutamate to glutathione [24]. Figure 5A showed that the CPB cell viability was 95% in the presence of 2.0 mM BSO. However, viral production was significantly decreased when cells were treated with BSO at 2.0 mM, indicating that glutathione synthesis was required for efficient ISKNV replication (Figure 5B). Glutathione is a reduced compound (reduced glutathione, GSH) and serves as a major cellular antioxidant via metabolic inter-conversion with oxidized glutathione (GSSG) [25]. Figure 5C and 5D showed that the amounts of GSH and GSSG in CPB cells infected with ISKNV were increased. GSH ethyl ester (GSHe) is a GSH analog that can be transported across the cell membrane and is converted to free GSH [26, 27]. In the presence of lower concentrations of GSHe (0.1–1 mM) in the cell medium, the yield of ISKNV was enhanced in CPB cells (Figure 5E). Taken together, these results suggested that ISKNV multiplication needed a certain amount of GSH and glutamine supplied substrate for GSH synthesis.

## DISCUSSION

Viruses have evolved to manipulate host cellular metabolism to support their energetic and biosynthetic requirements for successful replication. Several viruses reportedly alter glutamine metabolism to satisfy the biosynthetic and energy needs for their replications [10–12]. Our transcriptomic results showed that glutamine metabolism in CPB cells is greatly altered during ISKNV infection. We confirmed that ISKNV replication was inhibited in glutamine-free medium and was rescued by addition of glutamine. Furthermore, ISKNV replication in glutamine-depleted cells was substantially rescued by supplementation with TCA cycle intermediates, including α-KG, OAA, pyruvate, and citric acid. This suggests that in ISKNV infected cells, glutamine was converted to α-KG, which enters the TCA cycle (Figure 1). We also found that the anaplerosis efficiency of citric acid was significantly higher than those of α-KG, OAA, and pyruvate. The importance of citric acid generated by glutamine metabolism during ISKNV replication needs to be examined further.

Glutamine can be metabolized to α-KG to provide ATP through glutaminolysis [28]. Indeed, Chambers *et al*. [10] found that glutamine is a crucial nutrient for ATP generation. For example, addition of extra glucose to glutamine-depleted medium did not rescue ATP production during HCMV infection, indicating that the loss of ATP was due to the lack of glutamine. ATP is an energy source for the binding, maturation, assembly, and budding processes of many enveloped viruses [29]. It is noteworthy that ISKNV ORF123 encodes an ATPase, suggesting ISKNV may require extra ATP for efficient replication. We found that intracellular ATP levels were higher in ISKNV-infected cells than in mock-infected cells, and extracellular ATP increased ISKNV production. Moreover ATP levels and ISKNV yield in glutamine-repleted medium were higher than in glutamine-depleted medium, indicating that glutamine might directly provide ATP for ISKNV multiplication.

Our results also showed that glutamine affects viral transcription, protein translation and virion maturation. However, we could not determine whether glutamine affects all three of those steps or mainly the transcription step, since transcription step is situated upstream of protein translation and virion maturation. It is noteworthy that transcription of viral immediate early genes and early genes was not decreased (even a little bit increased) at 24 hpi in CPB cells cultured in glutamine-depleted medium. By contrast, transcription of a late gene (MCP gene) was inhibited throughout the course of the infection, indicating that glutamine might activate or suppress expression of viral immediate early genes, early genes and late genes via different mechanisms that remain to be elucidated. Interestingly, the pattern of glutaminase gene transcription was similar to that of viral immediate early genes and early genes, which are usually involved in the regulation of viral replication. Although the underlying mechanism is unknown, it is likely that under glutamine-depleted conditions, ISKNV alters not only cellular metabolism but also viral gene expression in order to compensate for the lack of glutamine.

Glutathione plays a prominent role in the defense against oxidative stress. The redox status of cells depends on the ratio of GSH to GSSG. Glutamine can be converted to glutamate, which is a substrate for GSH synthesis [11]. Glutathione synthetase (GSHB) is essential for glutathione synthesis. Our transcriptomic results showed that GSHB was up-regulated at 24 hpi and 72 hpi in CPB cells infected with ISKNV. Furthermore, ISKNV replication was inhibited by BSO, which indicates that glutathione is required for efficient production of ISKNV. Cai *et al*. [30] found that influenza virus replication is suppressed by GSH within the range of 1–30 mM, while it's replication is enhanced when GSH concentration are reduced to 0 mM. In our study, ISKNV yield was increased when the GSHe concentration was 0.1–1 mM and was inhibited when GSHe levels were more than 1 mM. This indicates that ISKNV multiplication benefits from a low GSH concentration. The GSH/GSSG ratio is reportedly about 100:1 in the normal cells, but this ratio can be as low as 1:1 under oxidative stress conditions [31]. Viral infection is often associated with oxidative stress, as an oxidized environment may favor viral infection [33]. The addition of extra GSH to the culture may break the balance between GSH and GSSG and create a redox environment. In this study, 2–10 mM GSHe decreased ISKNV replication, while 0.1–1 mM GSHe increased the virus replication. This indicates there is an optimal redox environment for efficient ISKNV replication. It is therefore likely that glutathione is involved in oxidative stress activated by ISKNV infection.

In sum, glutamine appears to be required for efficient ISKNV multiplication, as it provides a source for TCA cycle replenishment, ATP generation, and glutathione synthesis. ISKNV replication was inhibited by BPTES and EGCG, suggesting they or their analogs may be promising compounds for prevention of ISKNV infection.

## MATERIALS AND METHODS

### Cell lines and virus strains

Chinese perch brain cells (CPB) were established in our lab [32] and were propagated and maintained at 28°C in Leibovitz's L-15 medium (GIBCO, USA) supplemented with 10% fetal bovine serum (GIBCO, USA). For the glutamine depletion studies, DMEM (GIBCO, USA) with 1 g/liter D-glucose (Sigma, USA) but lacking L-glutamine and sodium pyruvate was used. This medium was supplemented with 2% dialyzed fetal bovine serum (Hyclone, USA), 2 mM L-glutamine (GIBCO, USA) was added and used as replete medium. Infectious spleen and kidney necrosis virus isolate (ISKNV-QY) was isolated in our lab previously [32]. ISKNV was propagated in CPB cells at 28°C and its titer was determined by TCID_50_ assay. The virus was stocked at −80°C until use.

### Reagents and antibodies

Dimethyl-α-ketoglutarate (α-KG), (–)-Epigallocatechinmo nogallate (EGCG), oxaloacetic acid (OAA), pyruvate, Adenosine 5ʹ-triphosphate (ATP), citric acid-anhydrous, glutathione reduced ethyl ester (GSHe), and D-(+)-glucose were purchased from Sigma-Aldrich (USA). L-Buthionine sulfoximine (BSO) was purchased from Santa Cruz Biotechnology (USA). All of above chemicals were diluted with culture medium to the indicated final concentrations just before use. Bis-2-(5-phenylacetamido-1,3,4- thiadiazol-2-yl) ethyl sulfide (BPTES) (Sigma, USA) was solubilized in dimethyl sulfoxide (DMSO) to a stock concentration of 10 mM. Additional dilutions of BPTES were made in methanol and used at the indicated final concentrations.

### Glutamine starvation

CPB cells grown in replete medium were washed with PBS and infected with ISKNV at an MOI of 1 for 2 h. Subsequently, cells were washed three times with PBS and fed with replete medium or glutamine-depleted medium. For BPTES or EGCG treatment experiment, ISKNV-infected cells were fed with replete medium containing 10 μM BPTES or 10 μM EGCG. For TCA intermediate rescue studies, TCA cycle intermediates were added to the medium and the final concentration was 7 mM of α-KG, 4 mM of OAA, 4 mM of pyruvate, and 4 mM of citric acid, respectively.

### Cell viability assay

The cytotoxic tests of glutamine-depleted, EGCG, BSO, citric acid, and ATP in CPB cells were performed using the CellTiter 96^®^ Aqueous One Solution Cell Proliferation Assay (MTS assay) (Promega, USA). Briefly, cells were seeded (5 × 10^4^ cells/well) in 96-well plates and allowed to attach overnight. Subsequently, these cells were washed with PBS once before fed with 100 μl glutamine-depleted medium, or glutamine-depleted medium supplemented with EGCG (0, 0.1, 1, 5, 10, 50, 100 μM), citric acid (0–7 mM), or ATP (0–6 mM), or BSO (0, 0.25, 0.5, 1, 1.5, 2, 2.5, 3 mM). At 72 h post treatment, 20 μl of the combined MTS/PMS solution was added into each well, incubated for 3 hours at 28°C, and then cell viability was determined by recording the OD_490_ nm in an ELISA microplate reader (Infinite M200 Pro, Tecan, Switzerland). Replete medium was used as control.

### Apoptosis assay by flow cytometry

CPB cells were seeded (5 × 10^4^cells/well) in 25 cm^2^ culture flasks and allowed to attach overnight. After culture medium was removed, cells were washed with PBS and then cultured in replete medium or glutamine-depleted medium. At 72 h post treatment, the cells were washed twice with ice cold HBSS, resuspended in 200 μl of binding buffer, and stained with AnnexinV-FITC and propidium iodide (PI) following manufacturer's instruction of FITC Annexin V Apoptosis Detection Kit (BD sciences, USA). After incubation for 15 min in the dark at room temperature, the cells were subjected to flow cytometric analysis (BD FACSVerse flow cytometer). Finally, the percentages of the apoptotic cells were analyzed using BD FACSuite software.

### ATP quantitation and ATP rescued experiment

Intracellular ATP concentrations at 12, 24, 36, 48 h post the addition of exogenous ATP were measured following the instruction of ATP Colorimetric/Fluorometric Assay Kit (Sigma, USA). Briefly, CPB cells were seeded (5 × 10^4^ cells/well) in 96-well plates and allowed to attach overnight. After culture medium was removed, cells were washed with PBS, then lysed in 100 μl ATP assay buffer, and incubated on ice for 10 min. The collected mix was centrifuged at 12,000 g for 3 min at 4°C. Ten microliter supernatant was added to the reaction mix used for the determination. The samples from three duplicates in black plates with clear bottoms were analyzed by Infinite M200 Pro using the fluorescence (FLU, λ_ex_=535 / λ_em_=587 nm).

For determination of infected cells ATP levels, cells infected with ISKNV were incubated in replete medium or glutamine-depleted medium supplemented with ATP (0 or 4 mM). The plates were incubated at 28°C with 5% CO_2_. Intracellular ATP level was measured by ATP Colorimetric/Fluorometric Assay Kit and the copy number of ISKNV were measured by quantitative PCR at 24 and 48 hpi.

### Quantification of ISKNV copies

The supernatants of CPB cells infected with ISKNV were treated with proteinase K (Promega, USA) with the final concentration of 160 μg/ml for 2 hours at 56°C, followed by incubation at 95°C for 10 min, centrifugation at 10000 rpm for 10 min. The centrifuged supernatants were used for q-PCR determination by using Premix Ex TaqTM assay (Takara, China) in an ABI 7500 Real-time Detection System (Applied Biosystems, USA). The used primers and probe were listed in Table 2. The copy numbers of ISKNV was calculated by comparison to the standard curve as described previously [33].

**Table 2:** Primers used for quantitative RT-PCR

Gens names	Genebank numbers	Uses	Primer names	Sequences (5ʹ–3ʹ)
ISKNV-MCP	NC_003494.1	Quantification analysis	F	CAATGTAGCACCCGCACTGACC
			Probe	FAM-CACCAAACTGACCG CGGACTCGT-Eclipse
			R	ACCTCACGCTCCTCGCTTGTC
GLS	Sequence obtained from transcriptom	Transcription analysis	q-GLS-F	TCCTGCGGCATGTACGACTTCT
			q-GLS-R	CCAGCTTGTCCAGTGGAGGTGA
ISKNV-MCP	NC_003494.1		q-MCP-F	CAATGTAGCACCCGCACTGACC
			q-MCP-R	ACCTCACGCTCCTCGCTTGTC
ISKNV-ORF038	NC_003494.1		q-038-F	GGTGGGGCGTGTAAAGCAGG
			q-038-R	GCGGTTTACTTCAAACAGGTCGG
ISKNV-ORF023	NC_003494.1		q-023-F	GATGGGAATTGTCATTGGGTCTTG
			q-023-R	CACAGCGGGTGAAACGGAAA
ISKNV-ORF034	NC_003494.1		q-034-F	CGCACGCACAACGAGACCA
			q-034-R	TCCTCGGAGACGCCCAGTG
ISKNV-ORF093	NC_003494.1		q-093-F	GAGTGCATGTCGATATGGTGGCA
			q-093-R	TGGCGTAGTTGGGGTGTTGGA
18s RNA	AY452495.1		q-18s-F	CATTCGTATTGTGCCGCTAGA
			q-18s-R	CAAATGCTTTCGCTTTGGTC

### Transcription of genes during ISKNV infection

The transcriptional levels of GLS and MCP genes were evaluated by quantitative reverse transcription PCR assays (qRT-PCR). Total RNAs were isolated from the cells at 72 hpi with TRIzol reagent (Invitrogen, USA), and then treated with RNase-free DNaseI (Promega, USA). The RNA was dissolved in 20 μl of RNase-free water and stored at −70°C until used. The amount of 1 μg RNA was used for reverse transcription to cDNA using RevertAid™ First Strand cDNA Synthesis Kit (Fermentas, CAN). The qRT-PCR was carried out in an ABI 7500 Real-time Detection System (Applied Biosystems, USA) using Maxima SYBR Green/ROX qPCR Master Mix (Fermentas, CAN). All PCR amplification reactions were performed in a volume of 20 μl, containing 0.8 μl cDNA, 0.3 μl of each primer (10 μM), 2 × Master Mix 10 μl. PCR conditions were as follows: 10 min at 95°C, followed by 40 cycles of 15 s at 95°C, 30 s at 60°C and 15 s at 72°C. Then at 95°C for 15 s, 60°C for 1 min, 95°C for 15 s. Melting curve was used to confirm the specificity of qRT-PCR amplification. The gene expression level was analyzed using comparative threshold cycle method (2^–ΔΔCT^) with 18sRNA gene as an internal control. The primers have been listed in Table 2. All data were given in terms of relative mRNA.

### Immunofluorescence assay

Cells were fixed with methanol for 20 min at room temperature and rinsed with PBS twice. Cells were incubated with the primary anti-MCP sera (1:200) for 1 h at room temperature, followed by three PBS washes. Then cells were incubated with the secondary fluorescein isothiocyanate (FITC)-conjugated goat anti-mouse IgG monoclonal antibody (1:100) (CWBIO, China) for 1 h at room temperature, followed by DAPI (2, 4-diamidino-2-phenylindole) (Beyotime, China) staining at a concentration of 1 mg/ml for 2 min at room temperature and washed thrice with PBS. The FITC signal was detected with an inverted fluorescence microscope (Nikon, Japan), and the images were captured by a digital imaging system (Nikon, Japan).

### Western blot analysis

CPB cells were collected and lysed in RIPA buffer with 1 mM PMSF. Proteins were separated by 12% SDS-PAGE and transferred onto Immobilon P polyvinylidene difluoride membranes (Millipore, USA). Blots were incubated with the indicated primary antibody, anti-ISKNV MCP (1:1000 dilution), anti-tubulin (1:5000 dilution), and subsequently incubated with peroxidase-conjugated goat-anti-rabbit IgG (1:5000 dilution). Immunoreactive proteins were visualized by chemiluminescence using Thermo Scientific Pierce Western Blot ECL Plus (Thermo, USA).

### Transmission electron microscopy

CPB cells with the confluency of 80–90% were infected with ISKNV at an MOI of 1 and fed with replete medium, or glutamine-depleted medium for 72 h. The infected cells were fixed with 2.5% glutaraldehyde in 0.1 M phosphate buffer (pH 7.4) for 24 h at 4°C and then post-fixed in 0.1 M phosphate buffer containing 1% osmium tetroxide for 1 h. Ultrathin sections were stained with uranyl acetate-lead citrate and examined by a Philips CM10 electron microscopy.

### Statistical analysis

Data were reported as mean ± standard deviation and analyzed using one-way analysis of variance (one-way ANOVA) followed by Fisher's LSD test using SPSS 17.0. *p <* 0.05 was considered significant and indicated by an asterisk while *p <* 0.01 was indicated by a double asterisks in the Figures.
